# Geographic disparity beyond the physical distance: Heart transplant outcomes in patients living in states without a transplant program

**DOI:** 10.1016/j.jhlto.2025.100365

**Published:** 2025-08-20

**Authors:** Toyokazu Endo, Joshua Crane, Isabelle Lytle, Jaimin Trivedi, Michele Gallo, Siddharth Pahwa, Mark S. Slaughter, Erin M. Schumer

**Affiliations:** a Department of Cardiovascular and Thoracic Surgery, University of Louisville School of Medicine, Louisville, Kentucky; b University of Louisville School of Medicine, Louisville, Kentucky

**Keywords:** heart transplant, out of state, geographical disparity, listing, heart failure

## Abstract

**Background:**

In the United States, outcomes of adult heart transplant are not well studied in those living in states without an active transplant program.

**Methods:**

Adult heart transplant patients were identified using the United Network of Organ Sharing database (2014-2023). Two groups were formed: out-of-state (OOS) for those in states without a program and in-state (IS) for those with a program. The primary outcome is post-transplant survival. Secondary outcomes examine listing characteristics and patterns using the Center for Disease Control WONDER database.

**Results:**

The OOS group (14 states) had 1,561 patients, with Nevada having the highest proportion. Fewer non-White individuals and those with government-sponsored insurance programs were in the OOS group (*p* < 0.05). Additionally, more patients in the OOS moved out of their primary state residence at the time of transplant (9.3% vs 2%, *p* < 0.01). Most patients traveled to high-volume centers in neighboring states to be listed. There was no difference in waitlist outcome (*p* = 0.13), but post-transplant survival was slightly higher in the OOS group (*p* = 0.04). Fewer patients in the OOS group were listed relative to their state population and the heart failure mortality cohort compared to those in the IS group (*p* < 0.01).

**Conclusions:**

Overall, the outcomes for individuals living in states without a transplant program did not differ compared to those in states with a program. However, variations in listing characteristics and patterns suggest a potential geographical disparity. Policy changes are crucial to address these inequalities and improve access to heart transplants in states that lack a transplant program.

## Graphical abstract

**Figure:**
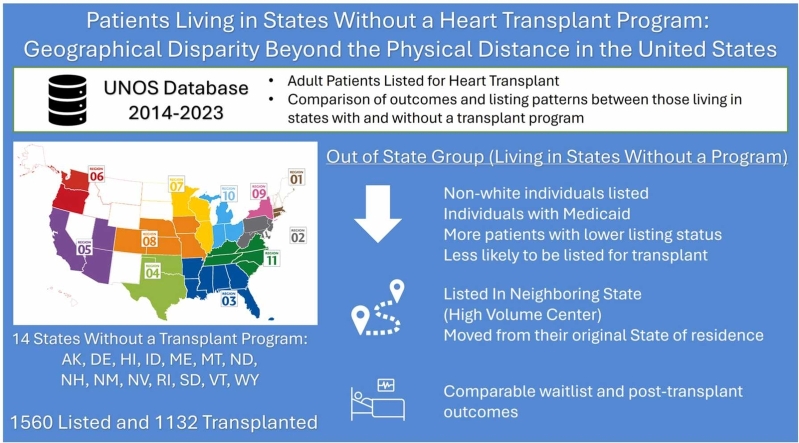

## Background

Over 6 million Americans have been diagnosed with heart failure, with the prevalence projected to rise by almost 2 million in the next 5 years.^1^ Treatments for heart failure range from guideline-directed medical therapy all the way to surgical interventions such as heart transplants and mechanical circulatory support devices.^2^ Heart transplantation is considered the gold standard for the treatment of end-stage heart failure in appropriate patients.^2^ There has been an 85.8% increase in heart transplantations from 2011 to 2022, indicating the growing demand. Thus, it is essential to address any gaps in the patient’s ability to access this life-saving procedure.

There are 145 active heart transplantation centers across the United States, with an average travel distance of less than 50 miles and 95% of recipients residing within a 5-hour radius of their designated center.^3^ There has been much discussion regarding whether the center’s distance affects both waitlist and post-transplant outcomes, and the literature is currently split.^4^, ^5^, ^6^, ^7^ In addition to distance, outcomes are also different based on the region of the United States, suggesting the presence of geographical disparity.^8^

Although numerous transplant programs exist in the United States, sometimes multiple within the same city, certain states lack an active heart transplant program. Data regarding patients' geographical locations and distances have been examined, but overall outcomes for those who must go out of state to be listed and transplanted remain unclear. In this study, we utilize a large national transplant database to investigate the listing characteristics and outcomes of individuals living in states with and without a transplant program.

## Methods

Adult patients ≥18 years who have been listed for a heart transplant were identified using the United Network of Organ Sharing (UNOS) thoracic transplantation database from 2014 to 2023. This period was chosen to account for the major US health care policy (Affordable Care Act) enacted in 2014.^9^ Two groups were created based on the patient’s primary state of residence at the time of listing. The UNOS database specifies the state of residence at the time of listing and also includes a state of permanent residence at the time of transplant. Those who resided in a state without a heart transplant program at listing were grouped under OOS and those living in a state with a program as in state (IS). The Organ Procurement and Transplantation Network (US Department of Health and Human Services) website was used to determine the presence of active heart transplant programs in each State.^10^ Patients residing outside the United States or in US-occupied territories (Puerto Rico, Guam, US Virgin Islands, etc.) were excluded. The primary outcome is to determine post-transplant survival between these 2 groups. The secondary outcome is to evaluate any differences in the listing characteristics, as well as waitlist survival between OOS and IS groups.

### Population and heart failure mortality data

Two separate analyses were conducted to determine the difference in the number of individuals listed between the groups. First, we calculated the trend of those listed for heart transplants to their respective home state’s population for each year of the study period. The proportion was multiplied by 10^6^ (million). Second, we calculated the proportion of those listed for heart transplants and the estimated prevalence of heart failure in each state. The prevalence of heart failure was estimated using the mortality rate.^11^ United States Center for Disease Control and Prevention (CDC) and the National Center for Health Statistics Mortality Data (CDC WONDER) were used to extract both the population data as well as heart failure (HF) mortality data during our study period.12., 13. The proportion of cumulative listing per state to the cumulative HF mortality was calculated. Furthermore, each state without a transplant program was compared with other states with a program in their respective UNOS regions.

### Statistical tests

Waitlist and transplant characteristics between the groups were compared using the chi-square test for categorical variables and the Kruskal-Wallis test for continuous variables. The log-rank (Kaplan-Meier) test was used to determine the overall waitlist and post-transplant survival. The population data was analyzed using parametric tests, including repeated measures ANOVA and the 2-proportions z-test. All statistical tests used an alpha of 0.05 and were conducted using Statistical Analysis Software (SAS, Cary, NC).

## Results

### States without a heart transplant program

There were a total of 14 states without an active adult heart transplant program in the United States (UNOS region 1: Maine, ME; New Hampshire, NH; Rhode Island, RI; Vermont, VT. Region 2: Delaware, DE. Region 5: Nevada, NV; New Mexico, NM. Region 6: Alaska, AK; Idaho, ID; Montana, MT; Hawaii, HI. Region 7: North Dakota, ND; South Dakota, SD. Region 8: Wyoming, WY).

### Waitlist characteristics and outcomes

**Figure 1:**
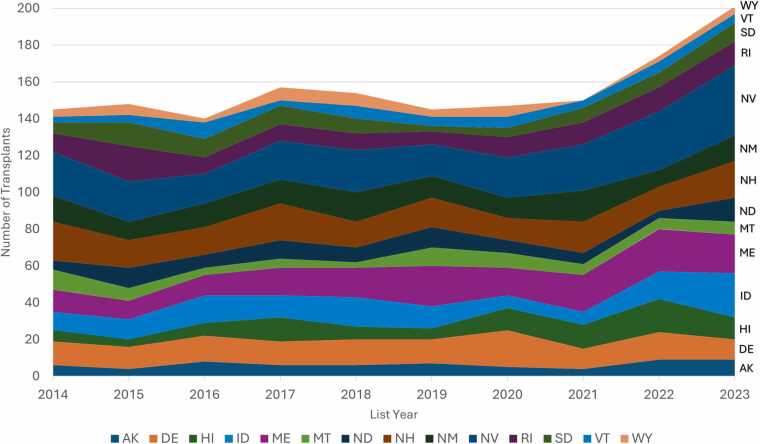
Number of patients listed from states without a transplant program. Alaska (AK)—64 (4.1%); Delaware (DE)—136 (8.7%); Hawaii (HI)—98 (2%); Idaho (ID)—129 (8.3%); Maine (ME)—165 (10.6%); Montana (MT)—67 (4.3%); North Dakota (ND)—82 (5.3%); New Hampshire (NH)—163 (10.4%); New Mexico (NM)—129 (8.3%); Nevada (NV)—240 (15.4%); Rhode Island (RI)—112 (7.2%); South Dakota (SD)—81 (5.2%); Vermont (VT)—52 (3.3%); Wyoming (WY)—43 (2.8%).

A total of 1,561 patients were listed in the OOS group. Nevada had the highest proportion of patients listed (240 [15.4%]), followed by Maine (165 [10.6%]), and Wyoming with only 43 (2.8%) patients (Figure 1). There was no significant difference in the number of individuals listed per year in all states within the OOS group except for Hawaii (*p* = 0.042).

Age and sex were similar in both groups, but there was a significant difference in terms of race, education level, and insurance distribution **(**Table 1). Significantly fewer non-White individuals were listed in the OOS group, including Black (10.1% vs 25.8%) and Hispanic (7.5% vs 9.8%) individuals (*p* < 0.001). More patients in the OOS group were more educated, and a higher proportion of individuals had some college education or degrees (*p* = 0.043). Furthermore, significantly more individuals had private insurance in the OOS group (54.5% vs 46.3%), while a higher proportion of Medicare (30.2% vs 35.9%) and Medicaid (10% vs 14%) were in the IS group (*p* < 0.001).

**Table 1:** Waitlist Characteristics

Variables	Out of state Median (IQR) *n* (%)	In state Median (IQR) *n* (%)	*p*-value
N	1,561	39,107	
Age	57 (46-64)	56 (46-63)	0.4410
BMI	27.4 (24-30.9)	27.8 (24.3-31.7)	0.0022
Gender (M)	1,136 (72.8)	28,808 (73.7)	0.4335
Race			<0.0001
White	1,140 (73)	23,375 (59.8)	
Black	157 (10.1)	10,105 (25.8)	
Hispanic	117 (7.5)	3,824 (9.8)	
Other	147 (9.4)	1,803 (4.6)	
Education level			0.0439
Less than HS	37 (2.45)	1,271 (3.4)	
HS or GED	544 (36)	14,296 (38)	
Some college	433 (28.6)	10,569 (28.1)	
Degree	499 (33)	11,532 (30.6)	
Insurance			<0.0001
Private	613 (54.5)	12,877 (46.3)	
Medicare	340 (30.2)	9,992 (35.9)	
Medicaid	112 (10)	3,892 (14)	
Other government	4 (0.4)	122 (0.4)	
Old initial status			<0.0001
1A	174 (24.9)	4,326 (25)	
1B	263 (37.6)	8,134 (47.1)	
2	262 (37.5)	4,824 (27.9)	
Initial status			0.0226
1	48 (5.8)	1,170 (5.5)	
2	190 (23)	5,618 (26.4)	
3	99 (12)	2,246 (10.6)	
4	289 (35)	7,560 (35.6)	
5	17 (2.1)	670 (3.2)	
6	183 (22.2)	3,994 (18.8)	
Diagnosis			<0.0001
Ischemic CM	401 (25.7)	10,627 (27.2)	
Idiopathic CM	462 (29.6)	13,622 (34.8)	
Other CM	154 (9.9)	3,024 (7.7)	
CHD	142 (9.1)	3,972 (10.2)	
Other	402 (25.8)	7,862 (20.1)	
Cigarette use	659 (24.2)	16,805 (43)	0.5492
Diabetes	420 (26.9)	12,026 (30.8)	0.0011
Prior cardiac surgery	573 (36.7)	15,203 (38.9)	0.1361
Dialysis	71 (6.3)	1,658 (6)	0.6268
Ventilator	23 (1.5)	780 (2)	0.1467
Inotropes	441 (28.3)	12,481 (31.9)	0.0023
MCSD			0.1368
LVAD	409 (26.2)	10,820 (27.7)	
RVAD	2 (0.1)	78 (0.2)	
TAH	5 (0.3)	139 (0.4)	
LVAD+RVAD	26 (1.7)	413 (1.1)	
ECMO	54 (3.5)	1,230 (3.2)	0.4865
IABP	108 (6.9)	3,873 (9.9)	<0.0001
Waitlist time	109 (21-394)	85 (19-329)	0.0006
WT death	176 (11.3)	4,349 (11.1)	0.8495
Transplanted	1,132 (72.5)	27,952 (71.5)	0.3711

Abbreviations: BMI, body mass index; CHD, congenital heart disease; CM, cardiomyopathy; ECMO, extracorporeal membrane oxygenation; GED, general educational development test; HS, high school; IABP, intra-aortic balloon pump; LVAD, left ventricular assist devices; M, male; MCSD, mechanical circulatory support devices; TAH, total artificial heart; WT, wait list.

The initial listing status also differed significantly in both the old and new allocation policies. More patients were listed as status 2 (37.5% vs 27.9%, *p* = 0.068) under the old policy and as status 6 after the allocation policy change (7.6% vs 4.5%, *p* < 0.001). Therapies at the time of listing, such as the use of ventilators, dialysis, and ECMO did not differ between groups (*p* > 0.05). Additionally, there was no difference in the proportion of individuals supported on durable mechanical circulatory support devices (*p* = 0.137). Though more patients in the IS group had intra-aortic balloon pumps (17.8% vs 19.4%, *p* < 0.001).

**Figure 2:**
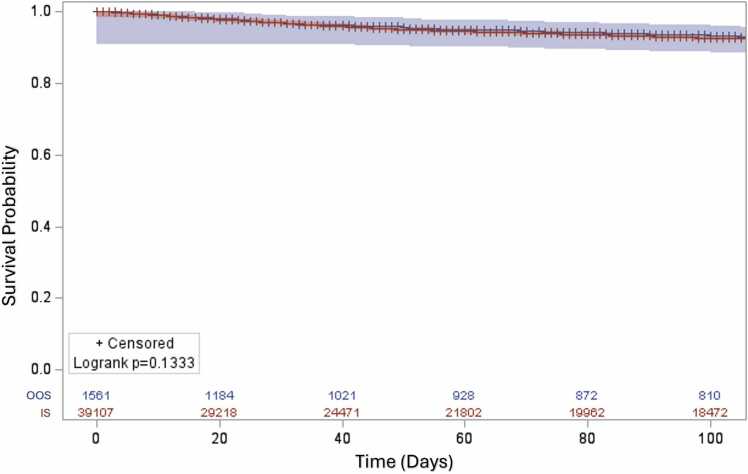
Waitlist mortality between OOS and IS group. There was no statistically significant difference in waitlist mortality between OOS and IS groups (*p* = 0.1333). (Blue—out of state [OOS], red—in state [IS]).

Though there were some significant differences in terms of patient characteristics, there was no difference in waitlist death in the univariate analysis (11.3% vs 11.1%, *p* = 0.85) and overall waitlist mortality **(**Figure 2, log-rank *p* = 0.13). However, the waitlist time was longer in the OOS group, with a median time of 109 days (21-394) compared to 85 days (19-329) in the IS group (*p* < 0.001).

### Transplant characteristics and outcomes

A total of 1,132 OOS and 27,952 IS patients were transplanted during the study period. The patient characteristics were similar to those of waitlist characteristics (Table 2). Though most of the patients were male and white in both groups, the distribution of race differed significantly with less non-White individuals in the OOS group (*p* < 0.001). Again, the distribution of insurance and education level was similar to that of waitlist characteristics.

**Table 2:** Transplant Characteristics

Variables	Out of state Median (IQR) *n* (%)	In state Median (IQR) *n* (%)	*p*-value
N	1,132	27,952	
Age	57 (46-64)	56 (46-63)	0.4396
Gender (M)	828 (73.1)	20,422 (73.1)	0.9503
BMI	27.3 (23.8-30.6)	27.5 (24-31.3)	0.0635
Race			<0.0001
White	818 (72.3)	16,862 (60.3)	
Black	107 (9.5)	6,947 (24.9)	
Hispanic	89 (7.9)	2,790 (10)	
Other	118 (10.4)	1,353 (4.8)	
Education level			0.0116
Less than HS	25 (2.3)	910 (3.4)	
HS or GED	374 (33.9)	10,133 (37.4)	
Some college	330 (30)	7,545 (27.8)	
Degree	375 (34)	8,531 (31.5)	
Insurance			<0.0001
Private	613 (54.5)	12,876 (46.3)	
Medicare	340 (30.2)	9,991 (35.9)	
Medicaid	112 (10)	3,892 (14)	
Other government	56 (5)	957 (3.4)	
Old initial status			0.0679
1A	247 (67.2)	7,148 (68.2)	
1B	115 (28.2)	3,046 (29.1)	
2	19 (4.7)	286 (2.7)	
Initial status			<0.0001
1	85 (11.7)	1,851 (10.6)	
2	323 (44.6)	8,861 (50.7)	
3	139 (19.2)	2,677 (15.3)	
4	119 (16.4)	3,123 (17.9)	
5	3 (0.4)	181 (1)	
6	55 (7.6)	779 (4.5)	
Diagnosis			<0.0001
Ischemic CM	278 (24.6)	7,435 (26.6)	
Idiopathic CM	346 (30.6)	9,805 (35.1)	
Other CM	112 (10)	2,270 (8.1)	
CHD	108 (9.5)	2,901 (10.4)	
Other	288 (25.4)	5,541 (19.8)	
Cigarette use	461 (40.7)	11,825 (42.3)	0.2913
Diabetes	301 (26.6)	8,383 (30)	0.0139
Cardiac surgery	275 (24.4)	5,942 (21.3)	0.0360
Dialysis	71 (6.3)	1,658 (6)	0.6272
Ventilator	16 (1.4)	489 (1.8)	0.3992
Inotropes	398 (35.2)	10,890 (39)	0.0111
MCSD			0.0165
LVAD	458 (40.7)	10,377 (37.3)	
RVAD	6 (0.5)	106 (0.4)	
TAH	6 (0.5)	205 (0.7)	
LVAD+RVAD	27 (2.4)	445 (1.6)	
ECMO	51 (4.5)	1,187 (4.3)	0.6655
IABP	167 (17.8)	5,409 (19.4)	0.0001
Waitlist time	60.5 (14-240)	48 (13-184)	0.0014
Moved out of state	105 (9.3)	570 (2)	<0.0001

Abbreviations: BMI, body mass index; CM, cardiomyopathy; CHD, congenital heart disease; ECMO, extracorporeal membrane oxygenation; GED, general educational development test; HS, high school; IABP, intra-aortic balloon pump; LVAD, left ventricular assist devices; M, male; MCSD, mechanical circulatory support devices; TAH, total artificial heart.

At the time of transplant, the final distribution of listing status did not differ significantly during the old allocation policy era (*p* = 0.067). However, there were some differences in the new allocation period (*p* < 0.001). More patients in the OOS group were transplanted as Status 1 (11.7% vs 10.6%), Status 3 (19.2% vs 15.3%), and Status 6 (7.6% vs 4.5%). The therapies at the time of transplant were similar to that of waitlist characteristics, with no difference in the proportion of individuals supported on a ventilator, on dialysis, or on ECMO (*p* > 0.05). A significantly higher proportion of individuals in the OOS were supported by a durable LVAD (40.7% vs 37.3%, *p* = 0.017).

**Figure 3:**
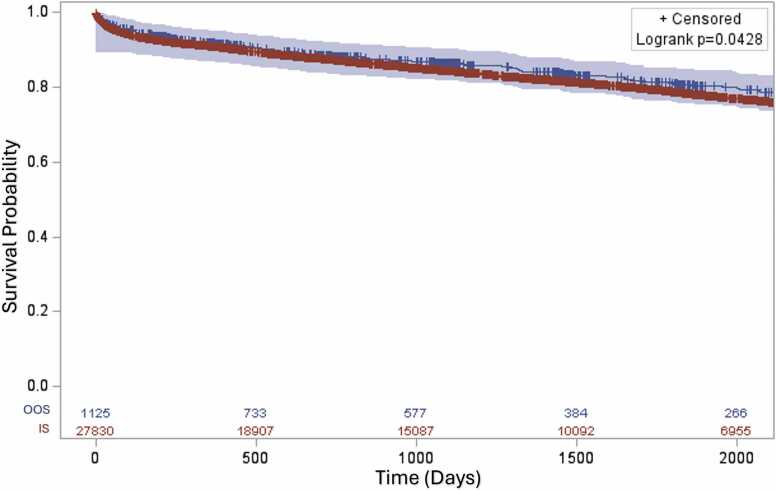
Post-transplant survival between OOS and IS group. Overall, there was a statistically significant difference in post-transplant survival between OOS and IS groups (*p* = 0.0428). (Blue—out of state [OOS], Red—in state [IS]).

Those that were transplanted from the OOS group had a higher median waitlist time of 60.5 days compared to 48 days in the IS group (*p* = 0.001). A total of 105 (9.3%) patients in the OOS group had a different permanent state address at the time of transplant, indicating that they had moved from their original state. Only 2% of patients moved in the IS group (p < 0.001). The overall post-transplant survival was slightly higher in the OOS group (Figure 3, *p* = 0.043), but there was no difference in survival between the different states within the OOS group (post-transplant survival between different states grouped according to their respective UNOS regions, Figure S1, *p* = 0.088). Additionally, the survival was compared based on each respective UNOS region between states with and without a transplant program. Except for UNOS region 6, all other OOS groups had comparable survival to the IS group in each UNOS region (OOS vs IS: region 1, *p* = 0.847; region 2, *p* = 0.855; region 5, *p* = 0.185; region 6, *p* = 0.027; region 7, *p* = 0.237; and region 8, *p* = 0.832, Figure S2).

**Figure 4:**
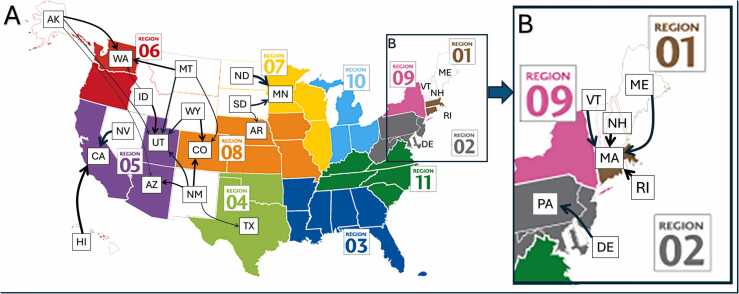
The states where patients living in states without a transplant program travel to be listed/transplanted. The majority of patients living in states without a transplant program traveled to their immediate neighboring state to be listed or transplanted (A). In regions 1 and 2, the majority of patients traveled to Massachusetts and Pennsylvania, respectively (B). The figure represents approximately 50% of the patients for each state and indicates which state they traveled to. The thickness of the arrows represents the degree of proportion (the thicker the arrow, the more patients compared to the thinner arrow). AK, Alaska; WA, Washington; ID, Idaho; HI, Hawaii; CA, California; NV, Nevada; UT, Utah; AZ, Arizona; MT, Montana; WY, Wyoming; CO, Colorado; NM, New Mexico; ND, North Dakota; SD, South Dakota; AR, Arkansas; MN, Minnesota; TX, Texas; ME, Maine; VT, Vermont; NH, New Hampshire; RI, Rhode Island; MA, Massachusetts; DE, Delaware; PA, Pennsylvania.

Source: Image adopted from UNOS website (https://optn.transplant.hrsa.gov/about/regions/).

More patients in the OOS group were transplanted at a high-volume center (83.4% vs 78.9%, *p* < 0.001).^14^, ^15^ Most patients in region 1 who did not have a transplant program traveled to Massachusetts, while those in region 2 went to Pennsylvania (Figure 4). Many patients from both Nevada and Hawaii were transplanted in California. Most states in the OOS group had patients transplanted in the neighboring states, except for those in Montana, most of whom traveled to Washington, Utah, or Colorado.

### Heart transplant listing per population and per heart failure mortality

**Figure 5:**
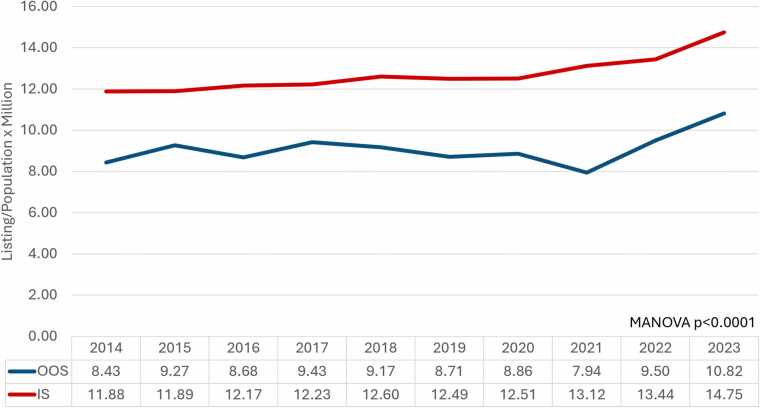
Heart transplants listed per population per year. The listing rate (Listing/Population × Million) increased steadily in both OOS and IS groups over the years. The population data were provided by CDC Wonder based on the US census data. The listing rate for the OOS group was significantly lower throughout the entire study period (*p* < 0.0001).

The proportion of individuals listed for heart transplants to their respective groups’ state population has increased over time in both OOS and IS (Figure 5). In 2014, the rate (listing/population× 10^5^) was 8.43 and 11.88 for OOS and IS, respectively. By the end of the study period, the rate increased to 10.82 and 14.75. The overall rate was significantly lower for the OOS group during our study period (*p* < 0.001).

The rate of listings per population varied for OOS and IS based on the UNOS region (Figure S3). The rates seemed similar in Region 2, with some notable differences in certain years. For regions 1, 5, 6, 7, and 8, the OOS group generally had a lower rate than their respective IS group. The rate per individual state per year is listed in Table S1 (Figure S4: United States Map: Rate of Listing Per Population).

**Figure 6:**
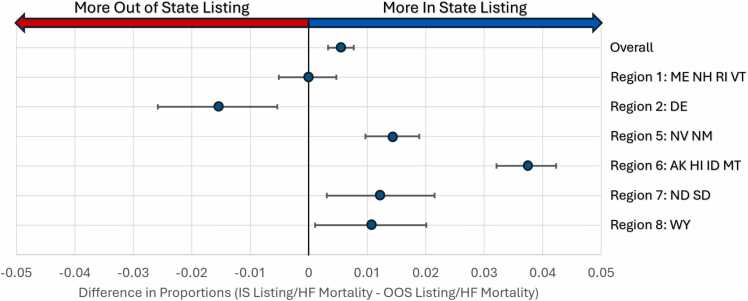
Difference in proportion of cumulative heart transplant listing to cumulative CDC heart failure mortality between 2014 and 2023. Overall, the proportion of cumulative listing to cumulative HF mortality for the OOS group was 4.6% (1,561/33,736) compared to 5.2% (39,107/746,730). More patients living in a state with a transplant program were more likely to be listed compared to those living in states without a program in regions 5, 6, and 7. Region 2 (Delaware) was the only region with more patients OOS listed compared to the IS group. (Dot representing the difference in proportions, with bars representing 95% CI). ME, Maine, NH, New Hampshire; RI, Rhode Island; VT, Vermont; DE, Delaware; NV, Nevada; NM, New Mexico; AK, Alaska; HI, Hawaii; ID, Idaho; MT, Montana; ND, North Dakota; SD, South Dakota; WY, Wyoming.

The relationship between heart failure mortality and listing was explored by comparing the proportion between the OOS and IS groups. Overall, the proportion of cumulative listing to cumulative HF mortality for the OOS group was 4.6% (1,561/33,736) compared to 5.2% (39,107/746,730) (Figure 6, *p* < 0.001). The OOS group and the IS group were further analyzed per UNOS region. In regions 5, 6, and 7, patients living in states with a transplant program had a higher chance of being listed for a transplant. Region 2 (Delaware) was the only region with more patients listed compared to the IS group.

## Discussion

The waitlist and transplant outcomes for individuals in states without a transplant program are comparable to those in states with a program. However, we have demonstrated that significant differences exist regarding patient characteristics. There was a disproportionate number of White individuals listed from the OOS group, along with significantly fewer individuals covered by government insurance, particularly Medicaid. Furthermore, the listing patterns for individuals in states without a program, although estimated, favor those in states with a transplant program. These findings suggest that disparities in access to care depend on the state of residence rather than the distance from a transplant center.^4^, ^6^

There is evidence that geographical disparity exists in the field of heart transplantation. Not every state's waitlist and post-transplant outcomes are equal, and policy can have a substantial impact on how organs are allocated based on geographical location and organ availability.^16^, ^17^ As we have demonstrated, some states in the Mountain West and Pacific regions must travel long distances to find a transplant center, while other states have multiple transplant centers located just a few miles apart.^16^ To address some of the differences in regional heterogeneity, policies such as extending the range of organ allocation to within 500 miles to medically urgent patients before offering to less urgent candidates have been introduced to create a broader network of organ sharing.^18^, 19., 20 However, the issue of geographical disparity in solid organ transplants extends beyond the heart; therefore, public policy related to this issue needs further attention, especially in states without an active transplant program.^20^, ^21^, ^22^, ^23^

When comparing the listing patterns in states without a transplant program, overall, there were significantly fewer patients listed compared to their respective state population and heart failure mortality. Several reasons could explain this, the first being that there may be insufficient structures to help list individuals who need a heart transplant at centers capable of performing such procedures. Most patients in the OOS group were listed and transplanted in their neighboring state, often at a high-volume center. It is impossible to speculate how specific health care organizations or heart failure cardiologists refer patients from OOS to transplant centers. However, our data suggest that individuals with private insurance, who have the means to travel and even relocate from their original state, may be more likely to do so.

The distribution of insurance also significantly differed in the OOS group, with notably fewer patients enrolled in Medicaid. Again, policies regarding interstate recognition, or even acceptance of government insurance for heart transplants, may further increase the number of individuals listed for heart transplantation. In states like Nevada and Montana, which do not have an active heart or lung transplant programs, State Medicaid does not cover such procedures, despite these states being part of the Medicaid Expansion under the Affordable Care Act.^24^, 25. Even if State Medicaid can cover heart transplants, it may require preauthorization, potentially delaying patient transfers and listings.^26^ Interstate recognition of government-sponsored insurance for congenital and pediatric care is currently pushed in the United States legislation and endorsed by the Society of Thoracic Surgeons.^27^ These policies serve as an excellent case study on whether such legislation, if passed, can enhance access for individuals in states lacking certain medical capabilities.

With the growing number of individuals afflicted with advanced heart failure, those living in states without an active transplant program may be at a disadvantage when it comes to being listed for heart transplantation. Although the OOS group showed better post-transplant survival, this might be due to notable differences in patient characteristics. Additionally, the listing pattern indicates that significantly fewer patients are being listed in the OOS group. While most of these patients are listed and transplanted at higher-volume centers in neighboring states, only those with the means or ability to do so can access these options. Since some states do not cover these procedures under their government-sponsored health insurance or require preauthorization, greater attention is needed to increase access to care in these regions. While the concept of regionalization in care is evident and may already have been implemented, it does not account for the unique interplay of interstate politics nor address geographical disparities.^28^, ^29^, ^30^ Further work is required in the field of heart transplantation and public policy to help meet the growing demand of the population for these life-saving procedures.

Once the life-saving procedure has been performed, it is also worthwhile to discuss the importance of follow-up care for patients who live out of state or far from the transplant center. Though there is some evidence that suggest that local cardiologists are coordinating care with the transplant center, the literature on how they manage these patients is limited.^6^ Telehealth has been proposed to improve access to care, but again, interstate medical and insurance policies may prohibit its use if they are in a different state.^31^, 32. With increased discussion regarding regionalization of care in heart transplantation, policies surrounding interstate recognition of insurance and licensure must be considered.

## Limitations

The study has several limitations due to its retrospective nature and reliance on a large database. One notable limitation is patient selection bias when examining outcomes. The OOS group comprised only a minority of the total heart transplants performed during our study period, meaning the statistical analysis compares 2 groups of vastly different sizes. We chose not to conduct a matched analysis because the primary objective of this manuscript is to present a side-by-side comparison of the characteristics and outcomes of individuals living in states without a transplant program. Although we have demonstrated a difference, it is possible that there are more non-White individuals with private insurance who have a higher educational level in the states that make up the OOS group. The CDC Wonder database lacks key information about non-White individuals in certain years and for certain states. Consequently, further elaboration on the state's demographic information cannot be conducted. Additionally, claims regarding patients' socioeconomic status and their potential means of travel have been corroborated in other studies using the UNOS database.^33^, ^34^

The population analysis of reported rates and proportions is intended only as an approximation to illustrate the relative nature of listing patterns. The CDC WONDER utilizes US census data, and both the total population and mortality rate may not be accurate. Furthermore, since there is no large, publicly available data reflecting the prevalence of heart failure, we used the mortality rate as a proxy to provide insight into how many individuals are affected by heart failure in each state. Finally, we were unable to analyze listing patterns based on race due to the unavailability of data in certain states and for specific years. While this retrospective study has multiple limitations, it does offer some insight into the current landscape of heart transplant listings and outcomes for those residing in states without an active program.

## Conclusion

Geographical disparity exists within the field of heart transplantation, and distance alone may not be the only factor. The characteristics of patients listed for transplant vary significantly from those in states without an active transplant program. Although the outcomes are comparable, further attention is needed to improve access to heart transplants in states without an active heart transplant program.

## Disclosure statement

None required—Data from the UNOS Database and CDC Wonder.

We thank the staff at the Department of Cardiovascular and Thoracic Surgery at the University of Louisville School of Medicine for their continued support in the research endeavors.

ISHLT Annual Meeting 2025, Boston, USA (Oral Presentation).

## Declaration of Competing Interest

The authors declare that they have no known competing financial interests or personal relationships that could have appeared to influence the work reported in this paper.

## Footnotes

Appendix A Supplementary data associated with this article can be found in the online version at doi:10.1016/j.jhlto.2025.100365.
