# Supplementary material for: Geographic disparity beyond the physical distance: Heart transplant outcomes in patients living in states without a transplant program
Source: JHLT Open. 2025 Aug 20;10:100365. doi: 10.1016/j.jhlto.2025.100365 (PMC12444177; doi:10.1016/j.jhlto.2025.100365)
Supplement: Supplementary file 1 — Supplementary material [file mmc1.docx]

**Supplement Figure 1: Post-Transplant Survival of All OOS States Groups in Their Respective UNOS Regions**

**
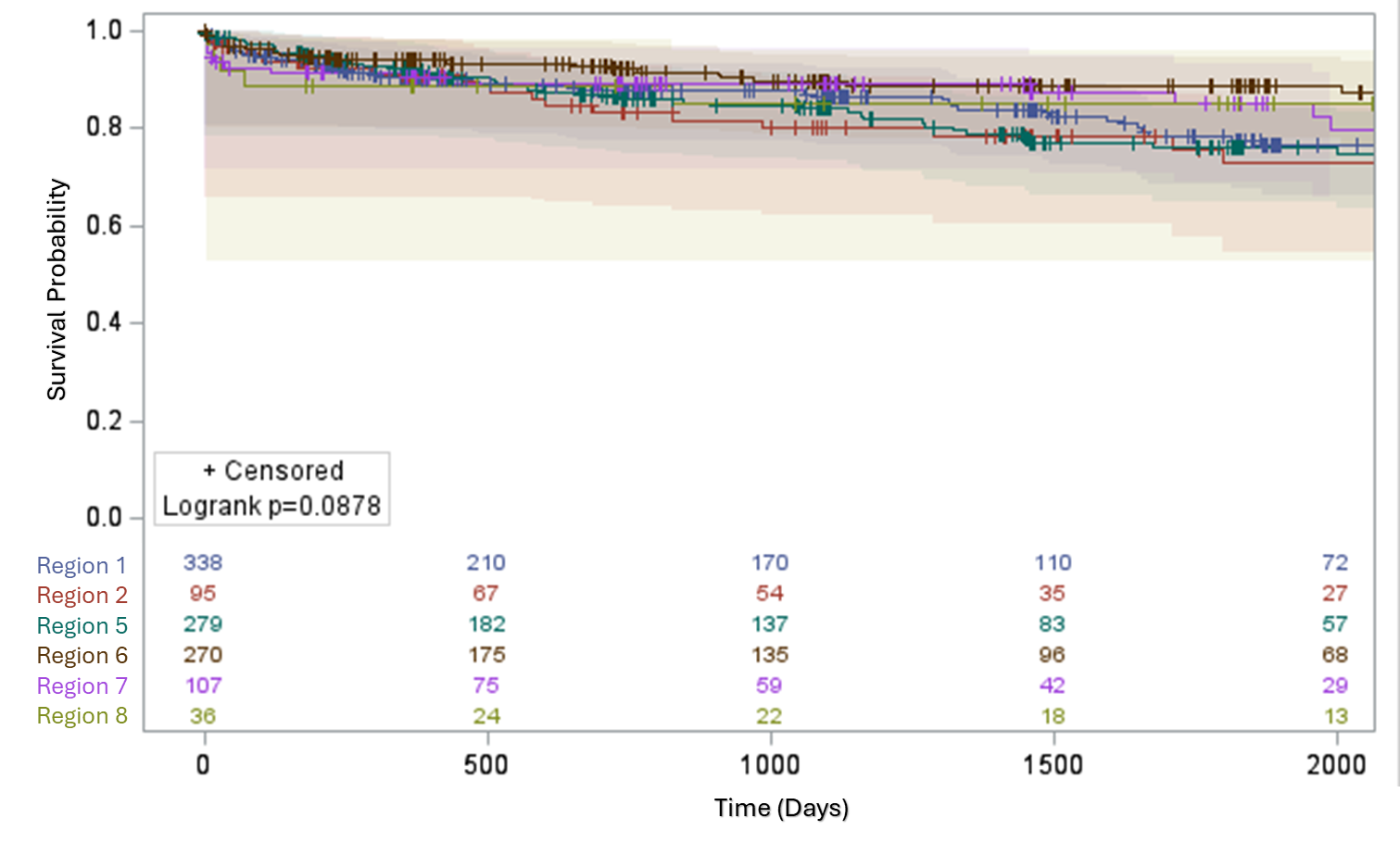

Supplement Figure 2: Post-Transplant Survival of OOS and IS Group for Each UNOS Region**

**
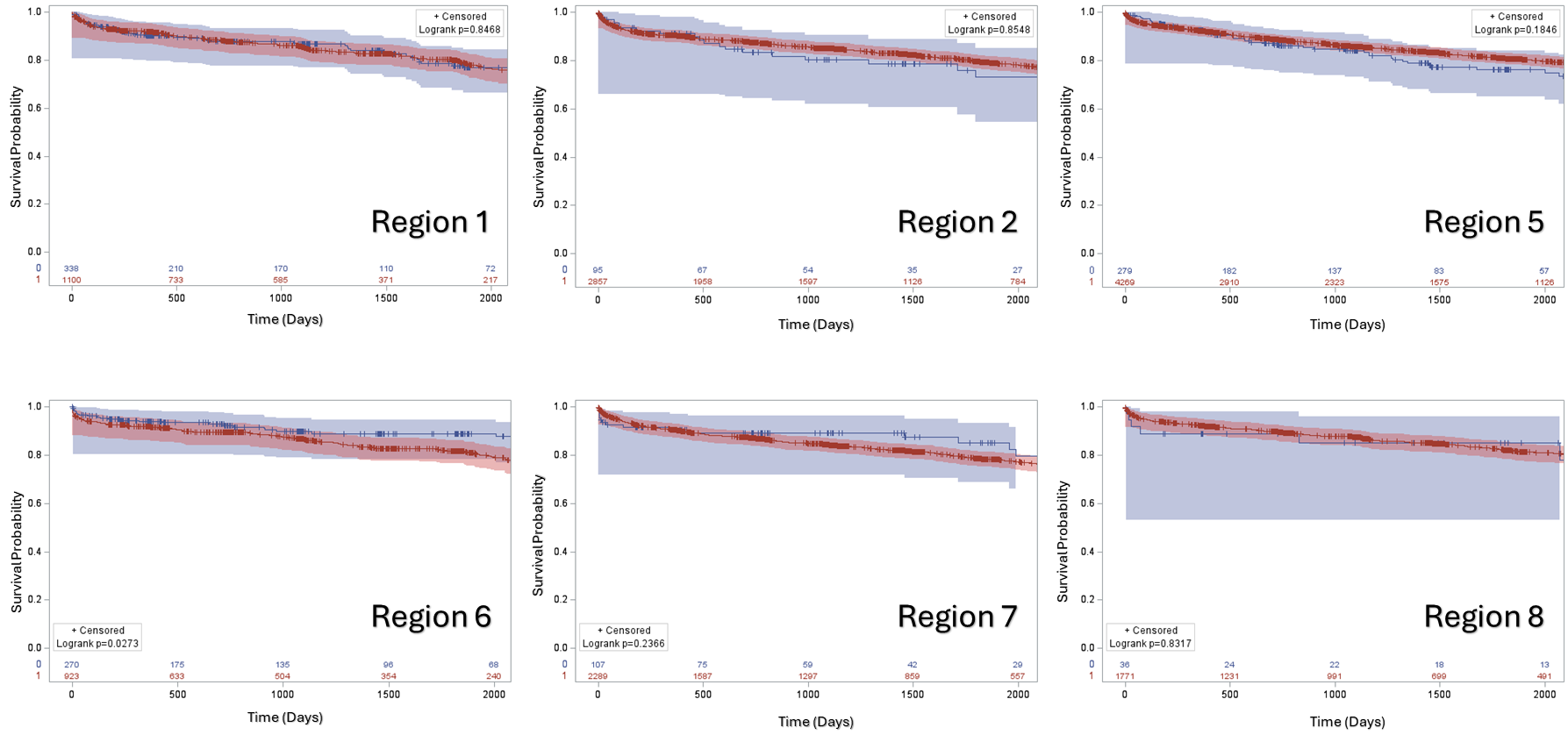
**

**Supplement Figure 3: Listing Per Population for OOS and IS Group per Each UNOS Region**

**
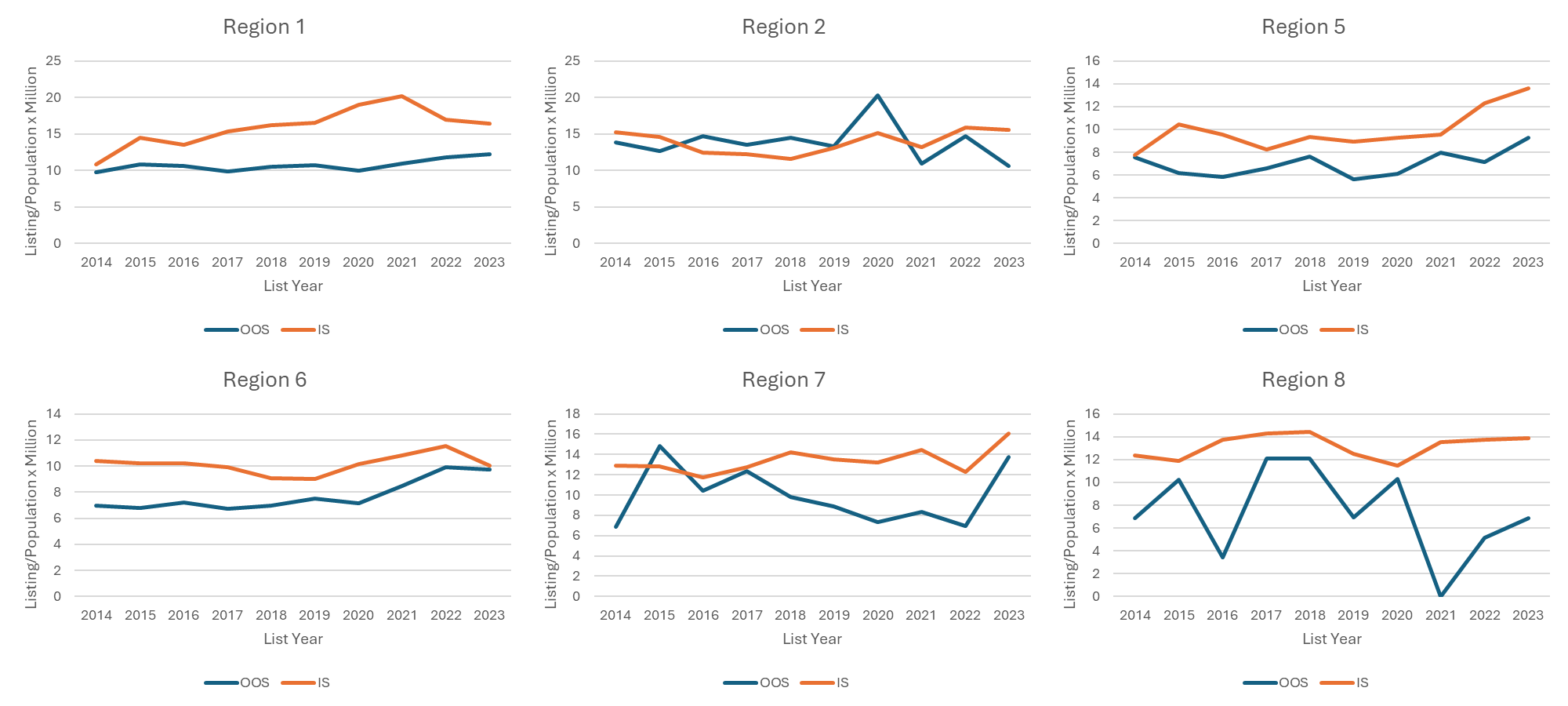
**

**Supplement Figure 4: Mean Listing Rate Per State Population**

**
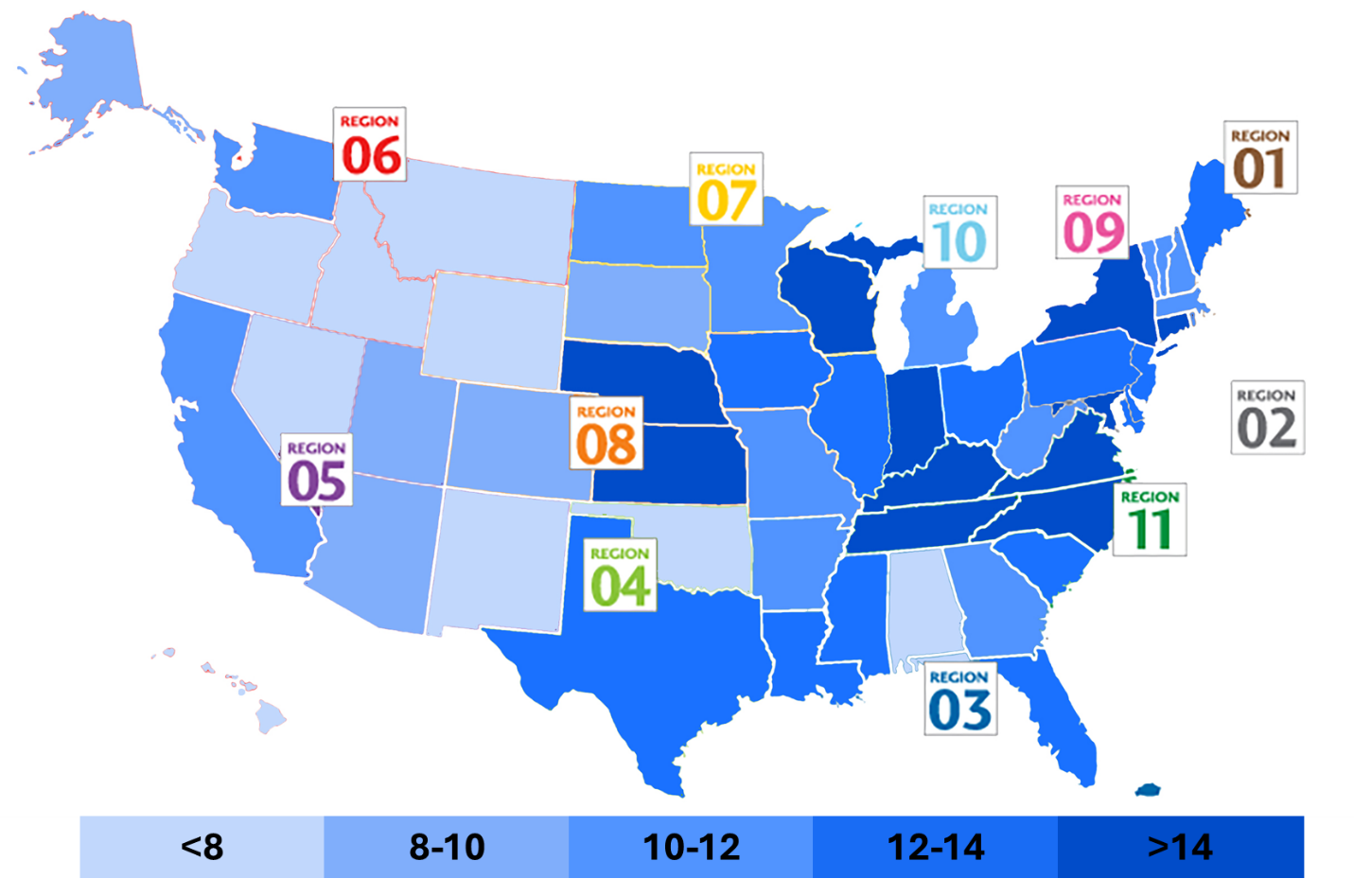
**

UNOS map with region depicting mean listing rate for each state from 2014-2023 (listed for heart transplant / state population x Million).

**Supplement Table 1: Heart Transplant Listing per State Population (per Million)**

| **State** | **2014** | **2015** | **2016** | **2017** | **2018** | **2019** | **2020** | **2021** | **2022** | **2023** | **Average** | **SD** |
| --- | --- | --- | --- | --- | --- | --- | --- | --- | --- | --- | --- | --- |
| Alabama | 9.49 | 7.61 | 7.81 | 7.18 | 7.98 | 6.12 | 7.11 | 7.54 | 8.87 | 7.63 | 7.73 | 0.93 |
| Alaska* | 8.14 | 5.42 | 10.78 | 8.11 | 8.14 | 9.57 | 6.84 | 5.46 | 12.27 | 12.27 | 8.70 | 2.50 |
| Arizona | 9.21 | 10.25 | 8.80 | 9.83 | 7.81 | 9.07 | 10.64 | 8.93 | 11.82 | 13.59 | 10.00 | 1.69 |
| Arkansas | 10.79 | 11.75 | 14.39 | 9.32 | 12.28 | 9.94 | 8.91 | 9.58 | 5.25 | 12.39 | 10.46 | 2.50 |
| California | 10.98 | 11.09 | 10.39 | 9.69 | 10.74 | 10.81 | 10.69 | 11.93 | 12.43 | 15.50 | 11.42 | 1.62 |
| Colorado | 6.16 | 8.61 | 8.30 | 7.13 | 9.31 | 7.47 | 7.40 | 7.57 | 8.73 | 11.23 | 8.19 | 1.40 |
| Connecticut | 12.23 | 18.66 | 15.66 | 16.16 | 20.99 | 23.00 | 27.27 | 28.57 | 21.51 | 18.52 | 20.26 | 5.12 |
| Delaware* | 13.89 | 12.69 | 14.70 | 13.51 | 14.48 | 13.35 | 20.27 | 10.96 | 14.73 | 10.66 | 13.92 | 2.65 |
| District of Columbia | 25.80 | 17.85 | 14.68 | 8.65 | 5.69 | 12.75 | 22.45 | 11.94 | 25.31 | 17.67 | 16.28 | 6.82 |
| Florida | 9.15 | 11.25 | 13.15 | 13.06 | 11.74 | 12.06 | 13.30 | 14.19 | 12.36 | 15.04 | 12.53 | 1.64 |
| Georgia | 8.72 | 10.18 | 8.54 | 7.77 | 9.03 | 8.10 | 10.18 | 11.76 | 13.29 | 21.22 | 10.88 | 4.02 |
| Hawaii* | 4.23 | 2.79 | 4.90 | 9.11 | 4.93 | 4.24 | 8.53 | 9.02 | 12.50 | 8.36 | 6.86 | 3.06 |
| Idaho* | 6.12 | 6.65 | 8.91 | 6.99 | 9.12 | 6.71 | 3.83 | 3.68 | 7.74 | 12.22 | 7.20 | 2.53 |
| Illinois | 13.12 | 15.16 | 13.05 | 14.61 | 12.95 | 13.73 | 13.58 | 14.99 | 12.95 | 15.70 | 13.98 | 1.04 |
| Indiana | 11.52 | 11.33 | 12.97 | 14.25 | 14.79 | 19.16 | 14.66 | 14.11 | 12.88 | 15.88 | 14.15 | 2.28 |
| Iowa | 12.87 | 14.41 | 15.95 | 17.17 | 11.09 | 11.09 | 9.17 | 14.41 | 13.75 | 9.67 | 12.96 | 2.66 |
| Kansas | 9.30 | 10.65 | 10.66 | 18.54 | 19.58 | 14.76 | 16.47 | 17.72 | 20.77 | 18.02 | 15.65 | 4.10 |
| Kentucky | 11.33 | 11.98 | 13.75 | 15.94 | 17.90 | 16.12 | 13.62 | 11.98 | 13.52 | 19.00 | 14.51 | 2.62 |
| Louisiana | 13.33 | 13.70 | 13.46 | 17.93 | 11.59 | 9.46 | 7.75 | 11.03 | 11.76 | 10.28 | 12.03 | 2.80 |
| Maine* | 9.02 | 7.52 | 8.26 | 11.23 | 11.95 | 16.37 | 11.11 | 14.57 | 16.60 | 15.05 | 12.17 | 3.34 |
| Maryland | 15.23 | 17.31 | 15.46 | 14.71 | 15.56 | 15.05 | 16.68 | 17.68 | 18.98 | 20.55 | 16.72 | 1.92 |
| Massachusetts | 9.34 | 10.30 | 11.30 | 14.43 | 11.45 | 10.16 | 10.73 | 11.74 | 12.32 | 14.28 | 11.61 | 1.68 |
| Michigan | 10.39 | 12.80 | 12.19 | 11.04 | 12.61 | 10.21 | 11.84 | 10.15 | 10.17 | 12.45 | 11.38 | 1.10 |
| Minnesota | 11.91 | 11.29 | 10.69 | 10.58 | 12.83 | 10.82 | 7.78 | 9.11 | 10.84 | 14.29 | 11.01 | 1.81 |
| Mississippi | 11.02 | 11.03 | 18.40 | 11.06 | 11.05 | 14.45 | 8.43 | 12.20 | 7.14 | 18.03 | 12.28 | 3.69 |
| Missouri | 12.20 | 8.38 | 8.53 | 8.83 | 9.79 | 11.73 | 11.87 | 13.78 | 14.24 | 14.20 | 11.36 | 2.33 |
| Montana* | 10.75 | 6.78 | 3.84 | 4.76 | 2.82 | 9.36 | 7.40 | 5.43 | 5.34 | 6.18 | 6.27 | 2.42 |
| Nebraska | 21.26 | 17.40 | 25.17 | 19.79 | 22.29 | 17.58 | 12.39 | 14.26 | 11.18 | 16.17 | 17.75 | 4.46 |
| Nevada* | 8.45 | 7.61 | 5.44 | 7.00 | 7.58 | 5.52 | 7.01 | 7.95 | 10.07 | 11.90 | 7.85 | 1.96 |
| New Hampshire* | 15.83 | 11.27 | 11.24 | 14.89 | 10.32 | 11.77 | 8.78 | 12.24 | 9.32 | 14.26 | 11.99 | 2.35 |
| New Jersey | 13.87 | 12.17 | 11.29 | 12.33 | 11.45 | 11.82 | 13.17 | 12.41 | 13.50 | 14.10 | 12.61 | 1.00 |
| New Mexico* | 6.71 | 4.80 | 6.25 | 6.23 | 7.64 | 5.72 | 5.22 | 8.03 | 4.26 | 6.62 | 6.15 | 1.19 |
| New York | 13.17 | 11.57 | 12.46 | 13.30 | 14.89 | 13.78 | 12.57 | 14.67 | 17.13 | 16.55 | 14.01 | 1.80 |
| North Carolina | 13.68 | 10.85 | 11.53 | 12.36 | 13.87 | 15.16 | 12.64 | 14.88 | 18.32 | 20.95 | 14.42 | 3.13 |
| North Dakota* | 6.76 | 14.53 | 9.24 | 13.24 | 10.53 | 14.43 | 9.15 | 7.74 | 5.13 | 16.58 | 10.73 | 3.79 |
| Ohio | 10.35 | 12.49 | 11.97 | 14.07 | 13.09 | 12.75 | 13.77 | 14.26 | 13.10 | 13.15 | 12.90 | 1.14 |
| Oklahoma | 11.09 | 5.88 | 7.90 | 8.14 | 8.12 | 7.83 | 10.80 | 6.27 | 6.72 | 5.67 | 7.84 | 1.88 |
| Oregon | 9.32 | 9.68 | 8.31 | 6.28 | 7.16 | 7.82 | 10.61 | 10.60 | 12.74 | 9.68 | 9.22 | 1.90 |
| Pennsylvania | 11.10 | 14.61 | 10.79 | 14.13 | 12.18 | 15.08 | 11.03 | 12.80 | 9.64 | 14.27 | 12.56 | 1.89 |
| Rhode Island* | 9.48 | 17.99 | 8.52 | 8.49 | 8.51 | 6.61 | 10.41 | 10.95 | 11.89 | 11.86 | 10.47 | 3.13 |
| South Carolina | 6.21 | 8.78 | 7.46 | 10.15 | 11.60 | 13.40 | 15.71 | 17.15 | 18.36 | 18.80 | 12.76 | 4.61 |
| South Dakota* | 7.03 | 15.14 | 11.55 | 11.50 | 9.07 | 3.39 | 5.60 | 8.93 | 8.79 | 10.88 | 9.19 | 3.34 |
| Tennessee | 15.12 | 10.45 | 14.88 | 14.74 | 16.40 | 15.96 | 15.39 | 18.06 | 17.30 | 17.68 | 15.60 | 2.16 |
| Texas | 16.06 | 13.58 | 13.85 | 12.05 | 12.82 | 12.90 | 10.59 | 13.51 | 13.42 | 13.54 | 13.23 | 1.39 |
| Utah | 3.06 | 10.01 | 9.50 | 5.16 | 9.49 | 6.86 | 6.46 | 7.79 | 12.72 | 11.70 | 8.28 | 2.98 |
| Vermont* | 4.79 | 6.39 | 14.41 | 4.81 | 11.18 | 8.01 | 9.63 | 6.20 | 9.27 | 7.72 | 8.24 | 3.01 |
| Virginia | 15.85 | 13.48 | 13.43 | 13.81 | 14.91 | 18.39 | 17.00 | 16.55 | 18.89 | 18.36 | 16.07 | 2.10 |
| Washington | 11.47 | 10.74 | 12.07 | 13.50 | 11.01 | 10.24 | 9.75 | 10.98 | 10.40 | 10.37 | 11.05 | 1.08 |
| West Virginia | 10.27 | 10.85 | 9.83 | 11.56 | 13.29 | 10.60 | 12.33 | 11.22 | 11.83 | 11.30 | 11.31 | 1.02 |
| Wisconsin | 13.72 | 11.96 | 11.59 | 13.11 | 16.86 | 15.97 | 18.17 | 19.17 | 13.07 | 18.27 | 15.19 | 2.83 |
| Wyoming* | 6.85 | 10.24 | 3.42 | 12.08 | 12.12 | 6.91 | 10.30 | 0.00 | 5.16 | 6.85 | 7.39 | 3.90 |

**Supplement Table 2:**

|  | 2013-2020 | | | 2021-2023 | | |
| --- | --- | --- | --- | --- | --- | --- |
| **STATE** | **Population** | **Deaths** | **Listed** | **Population** | **Death** | **Listed** |
| AL | 34158991 | 17786 | 260 | 15222641 | 7506 | 122 |
| AK | 5156994 | 576 | 42 | 2199662 | 320 | 22 |
| AZ | 49378654 | 5230 | 463 | 22066857 | 3211 | 253 |
| AR | 20999251 | 6682 | 232 | 9139260 | 3330 | 83 |
| CA | 275171334 | 50106 | 2924 | 117232371 | 26188 | 1557 |
| CO | 39222158 | 6101 | 305 | 17529605 | 2678 | 161 |
| CT | 25047157 | 7129 | 479 | 10848978 | 3825 | 248 |
| DE | 6723296 | 1343 | 99 | 3053670 | 848 | 37 |
| DC | 4827283 | 493 | 74 | 2020825 | 200 | 37 |
| FL | 146271782 | 27986 | 1753 | 66636677 | 13549 | 924 |
| GA | 72898868 | 23283 | 651 | 32741669 | 11305 | 506 |
| HI | 9950628 | 1875 | 55 | 4316887 | 650 | 43 |
| ID | 12057663 | 2842 | 83 | 5804682 | 1133 | 46 |
| IL | 89344568 | 28868 | 1228 | 37803190 | 14051 | 550 |
| IN | 46695456 | 13408 | 659 | 20501221 | 7056 | 293 |
| IA | 21986205 | 4249 | 288 | 9600600 | 2841 | 121 |
| KS | 20374698 | 5934 | 291 | 8812278 | 2268 | 166 |
| KY | 31143038 | 10959 | 448 | 13547858 | 4469 | 201 |
| LA | 32640489 | 14698 | 407 | 13788037 | 6570 | 152 |
| ME | 9359560 | 2327 | 101 | 4153309 | 746 | 64 |
| MD | 42195632 | 6165 | 663 | 18510042 | 2827 | 353 |
| MA | 47899654 | 14074 | 532 | 20968096 | 6159 | 268 |
| MI | 69672391 | 24141 | 807 | 30122185 | 9483 | 329 |
| MN | 38951478 | 7867 | 422 | 17162489 | 2958 | 196 |
| MS | 20888703 | 10352 | 255 | 8829712 | 4222 | 110 |
| MO | 42769221 | 16335 | 436 | 18542300 | 7617 | 261 |
| MT | 7361201 | 2392 | 48 | 3359950 | 852 | 19 |
| NE | 13406113 | 3650 | 260 | 5909994 | 1710 | 82 |
| NV | 20920848 | 2956 | 145 | 9515939 | 1250 | 95 |
| NH | 9417455 | 2620 | 113 | 4186277 | 1293 | 50 |
| NJ | 62519382 | 16377 | 769 | 27819670 | 8220 | 371 |
| NM | 14638342 | 2790 | 89 | 6343592 | 1403 | 40 |
| NY | 137469252 | 21407 | 1801 | 59084280 | 9026 | 952 |
| NC | 71879500 | 19875 | 926 | 32085626 | 10182 | 580 |
| ND | 5297202 | 1296 | 59 | 2338135 | 624 | 23 |
| OH | 81552327 | 25333 | 1031 | 35322010 | 11904 | 477 |
| OK | 27524647 | 6013 | 235 | 12060263 | 2510 | 75 |
| OR | 28885414 | 6900 | 244 | 12719650 | 2885 | 140 |
| PA | 89571779 | 32471 | 1138 | 38897747 | 15517 | 476 |
| RI | 7401337 | 1691 | 74 | 3285306 | 803 | 38 |
| SC | 35164997 | 9751 | 371 | 15846894 | 5288 | 287 |
| SD | 6106375 | 506 | 55 | 2724518 | 269 | 26 |
| TN | 47002847 | 9823 | 692 | 21153046 | 4146 | 374 |
| TX | 197651749 | 38418 | 2586 | 90060814 | 18568 | 1215 |
| UT | 21708813 | 6162 | 157 | 10136509 | 2895 | 109 |
| VT | 4374490 | 364 | 37 | 1940098 | 173 | 15 |
| VA | 59234877 | 16127 | 905 | 26041591 | 7926 | 467 |
| WA | 51769720 | 4980 | 582 | 23337358 | 2360 | 247 |
| WV | 12724179 | 3891 | 143 | 5328186 | 1652 | 61 |
| WI | 40571749 | 10797 | 588 | 17699402 | 4914 | 298 |
| WY | 4073900 | 962 | 36 | 1744241 | 393 | 7 |

**Supplement Table 2:**

| **CDC Wonder Data: Cumulative HF Death and Total Heart Transplant Listing** | | | | | | |
| --- | --- | --- | --- | --- | --- | --- |
| **2014-2020** | | |  | **2021-2023** | | |
| **Composite** | **Total Death** | **Total Listed** |  | **Composite** | **Total Death** | **Total Listed** |
| **Out of State** | **23504** | **1036** |  | **Out of State** | **10757** | **525** |
| **In State** | **507816** | **26005** |  | **In State** | **252016** | **13102** |
|  |  |  |  |  |  |  |
| **REGION 1: ME NH RI VT** | **Total Death** | **Total Listed** |  | **REGION 1: ME NH RI VT** | **Total Death** | **Listed** |
| **Out of State** | **7002** | **325** |  | **Out of State** | **3015** | **167** |
| **In State** | **21203** | **1011** |  | **In State** | **9984** | **516** |
|  |  |  |  |  |  |  |
| **REGION 2: DE** | **Total Death** | **Listed** |  | **REGION 2: DE** | **Total Death** | **Listed** |
| **Out of State** | **1343** | **99** |  | **Out of State** | **848** | **37** |
| **In State** | **59397** | **2787** |  | **In State** | **28416** | **1298** |
|  |  |  |  |  |  |  |
| **REGION 5: NV NM** | **Total Death** | **Listed** |  | **REGION 5: NV NM** | **Total Death** | **Listed** |
| **Out of State** | **5746** | **234** |  | **Out of State** | **2653** | **135** |
| **In State** | **61498** | **3544** |  | **In State** | **32294** | **1919** |
|  |  |  |  |  |  |  |
| **REGION 6: AK HI ID MT** | **Total Death** | **Listed** |  | **REGION 6: AK HI ID MT** | **Total Death** | **Listed** |
| **Out of State** | **7685** | **228** |  | **Out of State** | **2955** | **130** |
| **In State** | **11880** | **826** |  | **In State** | **5245** | **387** |
|  |  |  |  |  |  |  |
| **REGION 7: ND SD** | **Total Death** | **Listed** |  | **REGION 7: ND Sd** | **Total Death** | **Listed** |
| **Out of State** | **1802** | **114** |  | **Out of State** | **893** | **49** |
| **In State** | **47532** | **2238** |  | **In State** | **21923** | **1044** |
|  |  |  |  |  |  |  |
| **REGION 8: WY** | **Total Death** | **Listed** |  | **REGION 8: WY** | **Total Death** | **Listed** |
| **Out of State** | **962** | **36** |  | **Out of State** | **393** | **7** |
| **In State** | **36269** | **1580** |  | **In State** | **17114** | **791** |
